# Regression discontinuity threshold optimization

**DOI:** 10.1371/journal.pone.0276755

**Published:** 2022-11-16

**Authors:** Ioana Marinescu, Sofia Triantafillou, Konrad Kording

**Affiliations:** 1 School of Social Policy & Practice, University of Pennsylvania, Philadelphia, PA, United States of America; 2 Department of Mathematics and Applied Mathematics, University of Crete, Heraklion, Crete, Greece; 3 Departments of Bioengineering and Neuroscience, University of Pennsylvania, Philadelphia, PA, United States of America; Shandong University of Science and Technology, CHINA

## Abstract

Treatments often come with thresholds, e.g. we are given statins if our cholesterol is above a certain threshold. But which statin administration threshold maximizes our quality of life adjusted years? More generally, which threshold would optimize the average expected outcome? Regression discontinuity approaches are used to measure the local average treatment effect (LATE) and more recently also the Marginal Threshold Treatment Effect (MTTE), which shows how marginal changes in the threshold can affect the LATE. We extend this idea to define the problem of optimizing a policy threshold, i.e. selecting a threshold that optimizes the cumulative effect of the treatment on the treated. We present an estimator of the optimal threshold based on a constrained optimization framework. We show how to use machine learning (Gaussian process regression) for non-linear estimation. We also extend the estimation to a conservative threshold that is unlikely to produce harm, and we show how to include policy cost constraints. We apply these results to estimate an optimal tip-maximizing threshold for tip suggestions in taxi cabs Haggag (2014).

## 1 Introduction

Many real world optimization problems come in the form of a set of thresholds that need to be chosen. We give scholarships and access to better schools to students whose grades exceed certain thresholds [1], give drugs to patients whose lab tests exceed given values [2–4], give loans to people whose credit scores exceed some value [5], and show ads to unsuspecting web users whose likelihood to click on our ad exceeds a certain value [6]. In such cases, the decision maker faces an optimization problem: which level of the threshold will maximize the relevant welfare criterion? Threshold optimization has received little attention in the econometrics literature. We focus on Regression discontinuity (RD) designs where a threshold sharply defines treatment assignment.

RD designs are quasi-experimental designs that allow for local effect estimation [1]. In an RD design, an intervention is applied based on a cut-off on a running variable: units with a running variable value below the cutoff are assigned to the control condition, and units with a running variable value above the cutoff are assigned to the treatment condition (or vice versa). Assuming that the average properties of units just above and just below the threshold are similar, this allows estimation of the effect of the intervention at the threshold (Local Average Treatment Effect, LATE). These approaches are often implemented using local polynomial regression [7] and rely on continuity assumptions near the threshold (see [8] for a review of assumptions and methods). Alternative approaches are based on viewing RD design as a local randomization procedure [9, 10]. Regardless of the approach, the validity of this estimation weakens as we move to units away from the threshold, and the treatment effect estimation is only valid for a very specific subpopulation with running variable values very close to the threshold.

Recent works have gone beyond LATE estimation, trying to extend the validity of the effect estimation away from the threshold. [11] use difference-in-difference type assumptions to estimate the effect away from the threshold. [12] assume that the running variable is one of multiple noisy measures of a latent factor, and show that this allows for non-parametric estimation of the average effect away from the threshold. Angrist and Rokkanen [13] rely on the existence of a set of covariates that break the dependence of the policy on the running variable, and allow for extrapolation away from the threshold. [14] develop external validity tests based on the independence between types of compliance and potential outcomes. [15, 16] use RD policies with multiple cut-offs to estimate causal effects for different levels of the score variable.

Works mentioned sofar try to extend the validity of a LATE estimate beyond the threshold of the RD design. [17] also try to estimate how the LATE changes for small changes of the threshold; hence they evaluate the effect of marginally moving the policy threshold. This is called the Marginal Threshold Treatment effect (MTTE), and its estimation relies on some smoothness assumptions on the threshold. Building on the idea of estimating the effect of changing the threshold, we propose *optimizing* the threshold to maximize a welfare criterion (e.g. expected earnings over the population). For optimization, we need to make assumptions about the behavior of potential outcomes away from the threshold: first, we assume that linear extrapolation of potential outcomes is valid in a neighborhood around the threshold. Violations of this assumption introduce biases, and a highly relevant literature discusses related issues [18–20]. Our assumption is much stronger than continuity assumptions near the threshold; however, similar assumptions are often already necessary for the RD design to have statistical power. Second, we also assume that changing the threshold does not affect the treatment effect conditional on the running variable. Under these assumptions, we can extrapolate potential outcomes away from the threshold, and estimate the optimal threshold. While policy optimization has been explored for observational causal inference (e.g., [21, 22]), to the best of our knowledge, threshold optimization for RD designs has not been previously explored.

Our work contributes to the literature in the following ways: we introduce the welfare function in RD designs as an objective function for threshold optimization. We want to emphasize here that we can design meaningful welfare functions, primarily by assuming that we care about every individual being treated equally. We effectively want to maximize the population density weighted individual welfare. We derive estimates for optimal thresholds using local linear regression and Gaussian process regression. We obtain strategies to improve the threshold, including strategies that limit the risk of a negative local average treatment effect at the new threshold. These techniques could be a crucial piece for policy improvement. Finally, we apply the methods to estimate the tip-maximizing threshold for tip suggestions in taxi cabs [23].

Importantly, our calculations allow us to conceptualize RDD in new ways. We show that finding a zero treatment effect (on the relevant outcome that we want to maximize) in an RDD does not imply that the policy is ineffective but can indicate that the threshold is set optimally if the MTTE also changes at the threshold: in the canonical case of a positive treatment effect above the threshold, the LATE is zero for an optimally set threshold, and the treatment effect derivative is larger to the right of the optimal threshold than to the left of the optimal threshold. Many real-world thresholds are set by experts at levels that are likely to be quite adequate, so a zero treatment effect is a plausible outcome.

The rest of the paper is organized as follows: Section 2 briefly discusses the framework of sharp regression discontinuity designs, and Section 3 discusses recent results in the literature on the derivatives of LATEs. Our contribution begins with section 4, which defines the problem of optimizing policy thresholds in sharp and fuzzy RDDs and proposes methods of threshold optimization with local linear regression (Section 4.1) and Gaussian process regression (Section 4.2). Section 5 addresses risk aversion by discussing a modification of the threshold optimization problem so that the new threshold is unlikely to yield a negative LATE. In Section 7, threshold optimization is used to propose a new threshold for default tip suggestion in cab fares, that would maximize tip percentage. In Section 8 we summarize results and discuss pitfalls and future work.

## 2 Regression discontinuity designs

We first briefly discuss the framework of RD designs and how they can be used to obtain LATE estimates. For each unit *i* there are two potential outcomes, *Y*_*i*_(1) and *Y*_*i*_(0), corresponding to treatment and control, respectively. Let *T*_*i*_ denote the assignment for unit *i*, i.e. *T*_*i*_ = 1 if unit *i* is treated and 0 otherwise. In sharp RD designs, the assignment variable *T* is a deterministic piecewise function of a running variable *X*: *T*_*i*_ = *I*(*X*_*i*_ ≥ *c*). For each unit, only one of the outcomes is observed (they are either treated or control):

$$y_{i}=T_{i}Y_{i}\left(0\right)+\left(1-T_{i}\right)Y_{i}\left(1\right).$$

Let *μ*_1_(*x*) ≔ *E*[*Y*(1)|*X* = *x*] and *μ*_0_(*x*) ≔ *E*[*Y*(0)|*X* = *x*] be the conditional expectation of the outcome for treatment and control, respectively. The following assumption is necessary for identifying the LATE:

**Assumption 1** (Continuity). *μ*_1,0_ *are continuous in a small neighborhood around c*.

Under the monotonicity of the conditional Average Treatment Effects (ATEs) around *c*, the RD design allows the identification of *π*(*c*) = *E*[*Y*(1) − *Y*(0)|*X* = *c*)], as follows:

$$\begin{array}{c} \pi \left(c\right)=\;\underset{x\to c^{+}}{\text{lim}}\;E\left[Y_{i}|X_{i}=x\right]-\;\underset{x\to c^{-}}{\text{lim}}\;E\left[Y_{i}|X_{i}=x\right]=\;\underset{x\to c^{+}}{\text{lim}}\;\mu_{1}\left(c\right)-\;\underset{x\to c^{-}}{\text{lim}}\;\mu_{0}\left(c\right)=\mu_{1}\left(c\right)-\mu_{0}\left(c\right). \end{array}$$

Thus, the LATE *π*(*c*) is identifiable using a parametric or non-parametric estimator for *μ*_1,0_, as *μ*_1_(*c*) − *μ*_0_(*c*). Treatment assignment arbitrarily close to the threshold is considered random enough so that treated units right above the threshold are valid counterfactuals for non-treated units just below the threshold.

Typically, *μ*_1,0_ are estimated by applying a locally linear regression (LLR) model on data in a bandwidth *h* around *c*. The choice of the bandwidth is important, as an overly broad bandwidth will induce biases when the relationship between running variable and outcome are nonlinear. In practice, researchers may try different thresholds and test how the chosen bandwidth affects the obtained estimates [8]. For example, for LLR estimates, [24] propose a method for selecting an asymptotically optimal bandwidth in terms of the bias in the estimated LATE. Ideally, the chosen bandwidth must be narrow enough for the observations to be close enough to the cutoff to avoid large biases, and wide enough to provide adequate power.

Fuzzy RD designs (FRDD) handle settings where the threshold affects treatment assignment, but not deterministically. Let *T*_*i*_ be a binary variable indicating whether unit *i* has received treatment, and *T*^⋆^ = *I*(*X* ≥ *c*). As with the potential outcomes *Y*(*t*), the potential treatment status *T*(*t*^⋆^), indicates what an individual’s treatment status would be if *T*^⋆^ = *t*^⋆^ Let *f*(*x*) ≔ *P*(*T*_*i*_|*X*_*i*_ = *x*) be the probability that unit *i* with running variable *x* is treated. The probability of treatment changes discontinuously at the threshold, albeit not determinstically: lim_*x*→*c*^−^_ *f*(*x*) ≠ lim_*x*→*c*^+^_ *f*(*x*), 0 < *f*(*x*) < 1.

For every individual, we only observe one of the potential treatments *T*(0), *T*(1). Individuals can be classified based on their behavior towards treatment as: (a) compliers: *T*(0) < *T*(1), (b) defiers: *T*(0) > *T*(1) (c) always-takers: *T*(0) = *T*(1) = 1, and (d) never-takers: *T*(0) = *T*(1) = 0. LATE at the threshold *c* is the effect of treatment on compliers: *π*_*f*_(*c*) = *E*[*Y*(1) − *Y*(0)|*X* = *c*, *T*(0) < *T*(1)]. LATE identification then requires that, moving the threshold from *c* to *c* + *ϵ* will not create any defiers, and that there is at least one complier:

**Assumption 2** (Strong Monotonicity). *T*(0) ≤ *T*(1). *P*(*T*(0) > *T*(1)|*X* = *x*) *is strictly positive at X* = *c*.

[25] show that under the additional assumption of local independence, the running variable can be viewed as an instrumental variable, that affects the outcome only through treatment assignment at the threshold.

**Assumption 3** (Local Independence). *Y*(0) − *Y*(1) *is independent of T in a neighborhood of X* = *c*.

Under Assumptions 1, 2 and 3, the LATE at *X* = *c* can be identified as

$$\begin{array}{c} \begin{array}{cc} \pi_{f}\left(c\right) & =\frac{{\text{lim}}_{x\to c^{+}}E\left[Y_{i}|X_{i}=x\right]-{\text{lim}}_{x\to c^{-}}E\left[Y_{i}|X_{i}=x\right]}{{\text{lim}}_{x\to c^{+}}E\left[T_{i}|X_{i}=x\right]-{\text{lim}}_{x\to c^{-}}E\left[T_{i}|X_{i}=x\right]}\\ & =\frac{{\text{lim}}_{x\to c^{+}}\mu_{1}\left(x\right)-{\text{lim}}_{x\to c^{-}}\mu_{0}\left(x\right)}{{\text{lim}}_{x\to c^{+}}f\left(x\right)-{\text{lim}}_{x\to c^{-}}f\left(x\right)}\\ & =\frac{\mu_{1}\left(c\right)-\mu_{0}\left(c\right)}{{\text{lim}}_{x\to c^{+}}f\left(x\right)-{\text{lim}}_{x\to c^{-}}f\left(x\right)} \end{array} \end{array}$$ (2.1)

The local independence assumption implies that the treatment effect does not depend on the running variable directly or indirectly through confounding. This is often problematic in empirical applications, and does not allow the exploitation of derivative changes near the threshold employed in some recent work [18]. [26] shows that Eq (2.1) holds if we replace the local independence assumption with a continuity of selection into a type of individual:

**Assumption 4** (Local Smoothness). *The conditional means E*[*Y*(*t*)|*T*(0) < *T*(1), *X* = *x*] *and* *E*[*Y*(*t*)|*T*(0) = *T*(1) = *t*, *X* = *x*], *as well as the probabilities* *P*(*T*(0) < *T*(1)|*X* = *x*), *P*(*T*(0) = *T*(1)|*X* = *x*), *t* = 0, 1 *are continuous in x in a neighborhood of* *x* = *c*.

Assumption 4 can replace Assumptions 1 and 3 to obtain Eq (2.1). See [26] for a discussion on the behavioral assumptions that can lead to each of these assumptions. Importantly, removing the local independence assumption allows us to exploit changes of slope near the threshold.

## 3 Effect derivatives in RD designs

RD designs allow the unbiased estimation of the LATE at the predefined threshold under weak assumptions, and therefore have strong internal validity. Researchers are often interested in the external validity of the LATE and the effects of marginal threshold changes in the LATE. [17] show how to estimate the derivatives of the LATE under similarly weak conditions.

Specifically, let *S*(*x*, *c*) = *E*[*Y*(1) − *Y*(0)|*X* = *x*, *C* = *c*] be the average treatment effect of the sharp RD policy applied at threshold *c* on individuals with running variable *x*. Thus, the effect depends both on the value of the running variable *X* and on the implemented threshold *C*. For a constant threshold *C* = *c*, *π*(*X*) = *S*(*X*, *c*) shows how the treatment effect changes for different values of the running variable *X*. Respectively, *τ*(*C*) = *S*(*C*, *C*) shows how the LATE changes for different thresholds *C*. Notice that in the previous section, *π*(*x*) and *π*_*f*_(*x*) implicitly condition on *C* = *c*. In this section, we view treatment outcome as a function of both the value of the running variable and the value of the threshold.

[17] define the Treatment Effect Derivative (TED) as $\pi^{\prime }\left(c\right)=\frac{\partial S(X,c)}{\partial X}{|}_{X=c}$, and the Marginal Threshold Treatment Effect (MTTE) as $\tau^{\prime }\left(c\right)=\frac{\partial S(C,C)}{\partial C}{|}_{C=c}$. Thus, TED shows how the LATE varies for marginal changes in the running variable (and a given threshold), and the MTTE shows how the LATE varies for small changes in the threshold.

For fuzzy designs, let *S*_*f*_(*x*, *c*) = *E*[*Y*(1) − *Y*(0)|*X* = *c*, *C* = *c*, *T*(0) < *T*(1)] be the average treatment effect of the fuzzy RD design policy applied at threshold *c* on *complier* individuals with running variable *x*. TED and MTTE are similarly defined as $\pi_{f}^{\prime }\left(c\right)=\frac{\partial S_{f}\left(X,c\right)}{\partial X}{|}_{X=c}$, and the Marginal Threshold Treatment Effect (MTTE) as $\tau_{f}^{\prime }\left(c\right)=\frac{\partial S_{f}\left(C,C\right)}{\partial C}{|}_{C=c}$.

The authors provide conditions under which the TED and MTTE are identifiable. Specifically, non-parametric identifiability of TED for sharp RD designs is possible under the following assumption:

**Assumption 5** (Sharp RD TED). *E*[*Y*(*t*)|*X* = *x*] *is continuously differentiable in x in a neighborhood of x* = *c* *for t* = 0, 1.

Assumption 5 differs from Assumption 1 required for LATE identification in that it requires continuous differentiability at *x* = *c*, instead of continuity. However, most LATE estimators (parametric or non-parametric) require continuous differentiability at *x* = *c*. Under Assumption 1,

$$\begin{array}{c} \pi^{\prime }\left(c\right)=\mu_{1}^{\prime }\left(x\right)-\mu_{0}^{\prime }\left(x\right). \end{array}$$ (3.1)

For fuzzy RD designs, the function *π*_*f*_(*x*) additionally conditions on compliers, i.e. *T*(0) < *T*(1). The corresponding assumption for TED identifiability is the following:

**Assumption 6** (Fuzzy RD TED). *The conditional means E*[*Y*(*t*)|*T*(0) < *T*(1), *X* = *x*] *and* *E*[*Y*(*t*)|*T*(0) = *T*(1) = *t*, *X* = *x*], *as well as the probabilities P*(*T*(0) < *T*(1)|*X* = *x*), *P*(*T*(0) = *T*(1)|*X* = *x*), *t* = 0, 1 *are continuously differentiable in x in a neighborhood of x* = *c*.

Again, this assumption requires continuous differentiability at *x* = *c*, instead of just continuity (Assumption 4), which is required for LATE estimation. However, while not explicitly required for identification, the majority of parametric and non-parametric models used for estimation are continuously differentiable. Let *p*_*f*_(*c*) be the ratio of compliers at *X* = *c*. Under Assumptions 2 and 6, the TED for fuzzy RD designs is:

$$\begin{array}{c} \pi_{f}^{\prime }\left(c\right)=\frac{\mu_{1}^{\prime }\left(x\right)-\mu_{0}^{\prime }\left(x\right)}{p_{f}\left(c\right)}-\frac{p_{f}^{\prime }\left(c\right)\pi_{f}\left(c\right)}{p_{f}\left(c\right)}. \end{array}$$ (3.2)

Eq (3.1) can be viewed as a special case of Eq (3.2), where *p*(*c*) = 1 and *p*′(*c*) = 0. Eqs (3.1) and (3.2) can be used to identify the treatment effect derivative, which indicates how the effect would change for individuals with a slightly different value of the running variable. A large TED brings into question the external validity of the LATE estimator, since it implies that a small change in the running variable could have a large impact on the treatment effect.

TED captures the sensitivity of the LATE estimator to small changes in the running variable for a fixed threshold. MTTE answers the question: How would the LATE change for a small change in the implemented threshold? Naturally, since all of our samples come from a single RD design with a fixed threshold, additional assumptions are required to estimate MTTE: One has to make an assumption about how changing the threshold would affect potential outcomes. One common assumption is the **policy invariance assumption** [27], which states that *an individual’s outcome only depends on the treatment assigned to the agent, and not independently on the policy*. We use a weaker version of the policy invariance assumption, assuming mean independence instead of independence:

**Assumption 7** (Policy Invariance). *The potential outcomes for both treated and untreated are mean-independent of the threshold given the running variable*:

$$E\left[Y_{i}\left(t\right)|X_{i},C\right]=E\left[Y_{i}\left(t\right)|X_{i}\right]\;t=0,1.$$

This assumption may be unrealistic in many policies, in particular if the threshold is moved a lot. For example, if the threshold test score for acceptance at an elite school becomes lower, this may affect the treatment effect of admission for higher test score students due to peer effects. Additional examples are discussed in [27]. However, there are domains where the policy invariance assumption is more plausible: for example, the treatment effect of receiving a statin for patients with high cholesterol is not likely to change if patients with lower cholesterol also take the same statin. Moreover, to allow MTTE estimation, the policy invariance assumption only needs to hold *locally* at the threshold *c*.

Under (the local version of) Assumption 7, the MTTE is equal to the TED for sharp RD designs. Specifically, $\tau^{\prime }\left(c\right)=\pi^{\prime }\left(c\right)+\frac{\partial S(X,C)}{\partial C}{|}_{X=c,C=c}$. Local policy invariance implies $\frac{\partial S(X,C)}{\partial C}{|}_{X=c,C=c}=0$, hence, *τ*′(*c*) = *π*′(*c*). Similarly, for fuzzy designs $\tau_{f}^{\prime }\left(c\right)=\pi_{f}^{\prime }\left(c\right)+\frac{\partial S_{f}\left(X,C\right)}{\partial C}{|}_{X=c,C=c}$, and $\frac{\partial S_{f}\left(X,C\right)}{\partial C}{|}_{X=c,C=c}=0$, thus $\tau_{f}^{\prime }\left(c\right)=\pi_{f}^{\prime }\left(c\right)$. Thus, under local policy invariance, we can estimate how marginal changes of the threshold would affect the LATE at the (new) threshold.

## 4 Threshold optimization

The sign of the MTTE shows whether a marginal increase (decrease) of the threshold is likely to increase or decrease of the corresponding LATE. In many applications, we are interested in *optimizing* this threshold, i.e., we want to identify the threshold that maximizes the benefit of the interventions on the treated population. Let *p*(*c*) be the density of observations (individuals) at *x*, and that *x* ranges from 0 to *x*_*max*_. For sharp designs, we define the *welfare function* as the cumulative average treatment effect over all treated units:

$$W\left(t\right)=\int_{t}^{x_{max}}S(x,t)p(x)dx,$$

where *t* is the threshold at which the intervention is applied. Following the notation in Section 3, *S*(*x*, *t*) denotes the average treatment effect of individuals with a running variable of *x* when the policy is implemented at threshold *t*.

Without loss of generality, we assume the desired treatment effect is positive, hence we want to find the threshold that maximizes *W*(*t*). Assuming there are no spillover effects from the treated to the untreated, maximizing the welfare function corresponds to finding the threshold c* such that:

$$\begin{array}{c} c^{*}=\underset{t}{\text{arg}\;\text{max}}\;\int_{t}^{x_{max}}S(x,t)p(x)dx. \end{array}$$ (4.1)

Under the policy invariance assumption, *S*(*x*, *t*) = *π*(*x*)∀*t*, therefore the problem in Eq (4.1) becomes:

$$\begin{array}{c} c^{*}=\underset{t}{\text{arg}\;\text{max}}\;\int_{t}^{x_{max}}\pi (x)p(x)dx. \end{array}$$ (4.2)

Assuming that *π*(*x*), *p*(*x*) are continuous, and that *p*(*x*) is strictly positive in *x*, the first derivative of the welfare function is *W*′(*x*) = −*π*(*x*)*p*(*x*), and the threshold that maximizes the welfare function satisfies *π*(*x*) = 0. Thus, *the optimal threshold occurs at a value of the running variable where the LATE is zero*. Finding the optimal threshold is equivalent to finding the roots of *π*(*x*). The sign of the derivative left and right of the root indicates if the root corresponds to a local maximum or minimum. Eq (4.2) can then be solved analytically or numerically, given an estimate of the function *π*(*x*).

RD designs allow the estimation of *π*(*x*) only at *x* = *c*, since the identification of LATE is based on the assumption that treatment assignment is “as good as random” in an infinitesimally small neighborhood around *c*. Thus, the continuity of functions *μ*_1,0_ at the threshold suffices to allow estimation of *π*(*x*) locally at *c*, but not elsewhere. In practice, however, a functional form for ${\hat{\mu }}_{1,0}$ is obtained using the data in a bandwidth *c* − *h* < *X* ≤ *c* + *h*. Our approach is to use these estimates to extrapolate within this bandwidth. Notice that in this setting, we are using the bandwidth as a range where extrapolation of *π*(*x*) is valid. Thus, the local optimizer is the solution to the following bound constrained optimization problem, i.e.:

$$\begin{array}{c} c^{*}=\underset{t}{\text{arg}\;\text{max}}\;\int_{t}^{c+h}\pi (x)p(x)dx,\;c-h\leq t\leq c+h. \end{array}$$ (4.3)

In addition to the policy invariance assumption in [*c* − *h*, *c* + *h*], we assume the follwoing:

**Assumption 8** (Sharp optimization). *π*(*x*), *p*(*x*) *are continuous in* [*c* − *h*, *c* + *h*]. *p*(*x*) > 0 *in* [*c* − *h*, *c* + *h*].

Under Assumptions 7 and 8, it is straightforward to show that c* is a root of *π*(*x*), or lies on the border of the bandwidth.

For fuzzy RD designs, the welfare function is equal to the cumulative treatment effect on compliers:

$$\begin{array}{c} W_{f}\left(t\right)=\int_{t}^{x_{max}}S_{f}\left(x,t\right)p_{f}\left(x\right)p\left(x\right)dx \end{array}$$ (4.4)

Similar to the sharp RDD, we are interested in finding the optimal threshold c* in the interval [*c* − *h*, *c* + *h*]:

$$\begin{array}{c} c^{*}=\underset{t}{\text{arg}\;\text{max}}\;\int_{t}^{c+h}S_{f}\left(x,t\right)p_{f}\left(x\right)p\left(x\right)dx,\;c-h\leq t\leq c+h. \end{array}$$ (4.5)

Under the policy invariance assumption, Eq (4.5) becomes

$$\begin{array}{c} c^{*}=\underset{t}{\text{arg}\;\text{max}}\;\int_{t}^{c+h}\pi_{f}\left(x\right)p_{f}\left(x\right)p\left(x\right)dx,\;c-h\leq t\leq c+h. \end{array}$$ (4.6)

Similar to the sharp RD, we make the following additional assumptions:

**Assumption 9** (Fuzzy optimization). *π*_*f*_(*x*), *p*(*x*), *p*_*f*_(*x*) *are continuous in* [*c* − *h*, *c* + *h*]. *p*_*f*_(*x*), *p*(*x*) > 0 *in* [*c* − *h*, *c* + *h*].

Under assumptions 7, 9, the optimal threshold is a root of the treatment effect function, *π*_*f*_(*x*) = 0. If *π*_*f*_(*x*) does not have a root in [*c* − *h*, *c* + *h*], then the local optimum lies on one of the borders of the interval.

The global optima of Eqs (4.2) and (4.4) could lie outside the bandwidth around the implemented threshold, and can be very different from the local optima in the within-bandwidth versions in Eqs (4.3) and (4.6). However, we cannot safely extrapolate *π*(*x*) away from the bandwidth, without losing the benefits of the RD design, or without additional assumptions. Moreover, we argue that policy thresholds are typically implemented based on some prior evidence on the benefits/cost of the policy. We therefore expect that the global optimum should lie close to the implemented policy thresholds. Furthermore, we view policy design and optimization as an iterative process. In this process, the prediction of an improved outcome results in moving the threshold within a small neighborhood of the existing threshold (in analogy to trust region methods used in machine learning [28]); in turn, researchers can collect new data from the new policy, extending the data support for *π*(*x*) and allowing for further extrapolation.

Importantly, the threshold optimization approach presented here also allows a new interpretation to RDD estimates. Typically, a zero LATE is considered to imply an ineffective intervention. If LATE is zero for all values of the running variable, then the intervention is ineffective, and changing the threshold will not help (the MTTE is zero). However, a zero LATE can indicate an intervention for which utility is maximized: *zero LATE accompanied by a non-zero MTTE at the threshold is consistent with the threshold being set optimally to maximize outcomes*.

### 4.1 Estimation with local linear regression

In the previous section, we defined the problem of threshold optimization, and showed that the optimal threshold corresponds to a zero LATE (*π*(*x*) = 0). To identify the optimal threshold, we need to estimate *π*(*x*) = *μ*_1_(*x*) − *μ*0(*x*) in a neighborhood of *c* and identify the threshold that leads to the optimal value of the welfare function within this neighborhood.

Typically, *μ*_1,0_ are estimated using locally linear regression (LLR), which has been shown to have desirable bias properties at the threshold [7]. In the case of a rectangular kernel, this approach is equivalent to taking standard OLS estimates on both sides of the threshold. Different kernels could be used, but typically have little impact on the estimated effect [8]. Thus, we will focus on LLR with a rectangular kernel.

For a given bandwidth *h*, the regression model below the threshold is

$$\begin{array}{c} Y\left(0\right)=\alpha_{0}+\beta_{0}\left(X-c\right)+\varepsilon ,\;-h\leq X-c<0 \end{array}$$

while the regression model above the threshold is

$$\begin{array}{c} Y\left(1\right)=\alpha_{1}+\beta_{1}\left(X-c\right)+\varepsilon ,\;0\leq X-c\leq h, \end{array}$$

where *ε* follows a zero mean Gaussian distribution with standard deviation *σ*. Using OLS estimates for *α*_0,1_, *β*_0,1_, the treatment effect is a linear function of the running variable:

$$\begin{array}{c} \hat{\pi }\left(x\right)={\hat{\mu }}_{1}\left(x\right)-{\hat{\mu }}_{0}\left(x\right)={\hat{\alpha }}_{1}-{\hat{\alpha }}_{0}+\left({\hat{\beta }}_{1}-{\hat{\beta }}_{0}\right)\left(x-c\right). \end{array}$$

At the threshold *c*, we have the RDD treatment effect estimate which is equal to the difference of intercepts $\hat{\pi }\left(c\right)={\hat{\alpha }}_{1}-{\hat{\alpha }}_{0}$. The utility function has one global extremum at $\hat{\pi }\left(x\right)=0$, thus

$$\begin{array}{c} \hat{c^{*}}=c-\frac{{\hat{\alpha }}_{1}-{\hat{\alpha }}_{0}}{{\hat{\beta }}_{1}-{\hat{\beta }}_{0}}. \end{array}$$ (4.7)

**Fig 1:**
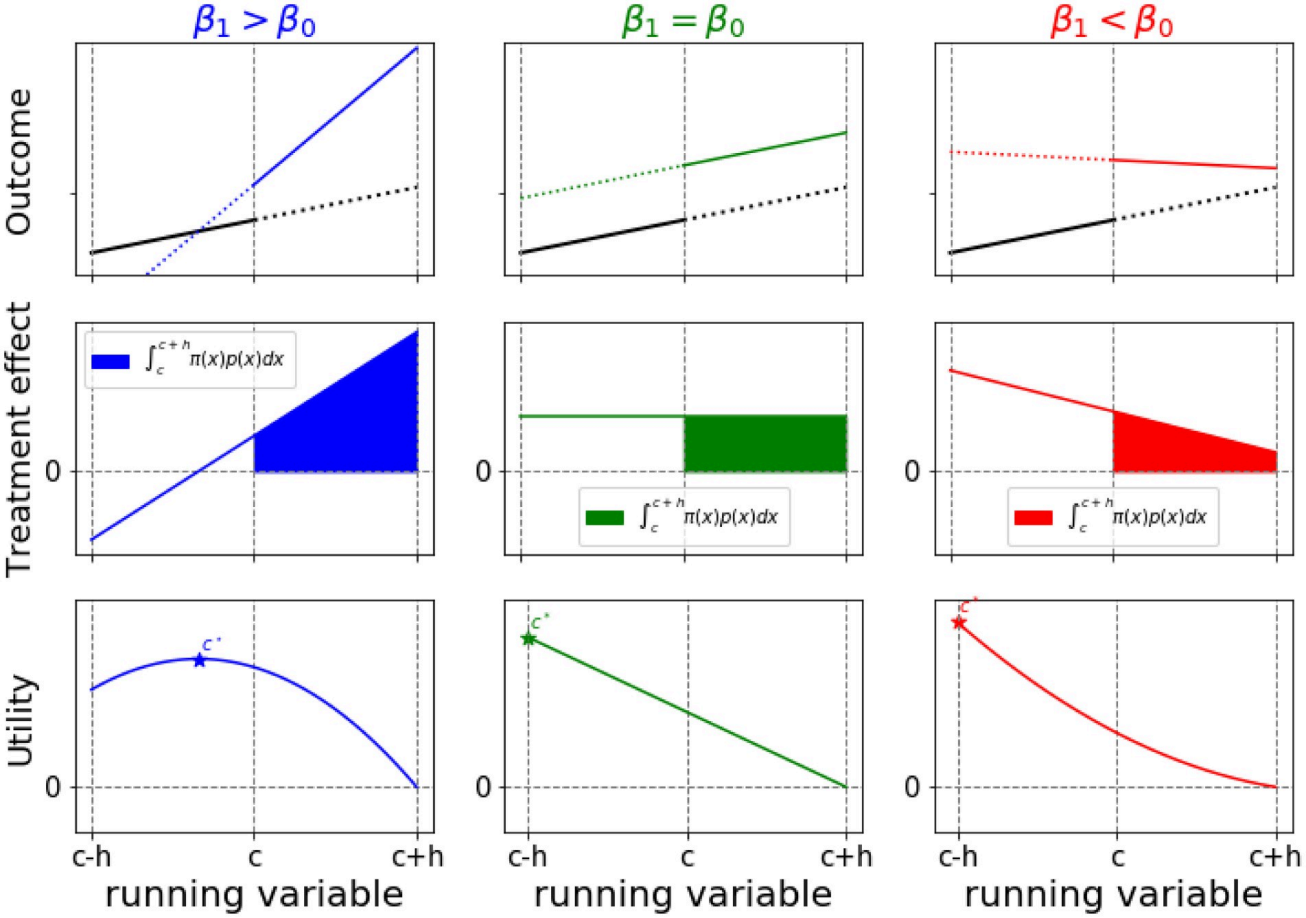
Optimizing the threshold for locally linear regression (LLR) models. (top panel) Possible LLR models for outcome vs running variable. The discontinuity at *c* corresponds to the LATE *π*(*c*). (middle panel) Treatment effect *π*(*x*) as a function of the running variable; the colored area corresponds to the utility. (bottom panel) Utility as a function of the chosen threshold, for uniform density of observations. The constrained optimization problem has one global optimal, either where *π*(*x*) crosses the x-axis (left column) or at the left boundary (middle, right column).

There are a range of different scenarios that matter for the threshold optimization problem in the context of LLR (Fig 1). Intuitively, we know that if the LATE is positive, we need to move the threshold to the left in order to treat more units, and if the LATE is negative we need to move it to the right. In Fig 1, we focus on cases where the LATE is positive, and vary the treatment effect derivative. The canonical case has a positive TED difference and a positive LATE (left panels in Fig 1). (Note that, if the TED were not higher right than left, the two lines would fail to cross.) However, there can be exceptions. For example, the LATE may be nonzero while the TED difference is zero. This may suggest that the treatment is always better (or worse) than the control and the threshold optimization problem becomes locally undefined (middle panels in Fig 1). Further, there may be some counterintuitive *π* where the LATE and the TED difference have opposite signs, indicating that the treatment stops being effective (or even becomes harmful) for large values of the running variable (right panels in Fig 1). In this paper we will generally analyze the canonical case in the left panels of Fig 1.

Within the bandwidth, if the treatment effect has a root *c*′ on the left of our observed threshold *c* (Fig 1, left column), then this root is the global maximum of the utility function. This is the canonical case where the LATE is zero at the optimal threshold c* and positive to the right of c*, and we further have *β*_1_ > *β*_0_, i.e. the treatment effect derivative is greater above c* than below c*. This shows that a zero LATE does not demonstrate that a policy is ineffective: a zero LATE with an increase in the treatment effect derivative is the sign of an optimally set threshold.

If the treatment effect does not have a root *c*′ on the left of threshold *c* (Fig 1, middle and right columns), the model suggests that the threshold should be moved to the left boundary (or even beyond). This yields a less trustworthy estimate, since we have less faith in the modeling assumptions as we move further and further away from the threshold. We later discuss more conservative estimates for threshold updating.

### 4.2 Non-linear estimation with Gaussian process regression

If we do not want to assume that the relationship between the running variable and the outcome is linear close to the threshold, we can use other strategies. For example, controlling for a higher-order local polynomial in the running variable has been popular. However, this method produces estimates that are particularly sensitive to the choice of polynomial degree [29]. Gaussian processes (GPs) refer to a class of approaches that offer an alternative way of setting up the nonlinear regression problem.

Recently, Branson et al. [30] proposed using Gaussian process regression (GPR) to model the conditional expectations *E*[*Y*(1)|*X* = *x*] and *E*[*Y*(0)|*X* = *x*] and estimate the LATE at the threshold *c* in RD designs. The authors show that GPR produces consistent estimates for LATE and can outperform state-of-the-art LLR methods, especially in terms of coverage.

GPR derives from the Bayesian idea that we should start with a prior belief over the functions we may encounter. Such a belief could for example be that smoother functions are more probable than less smooth ones. More specifically, we are interested in the function *f* that maps the running variable to the outcomes, so we need a prior belief *p*(*f*) about the possible functions *y* = *f*(*x*), where *y* can denote either of the potential outcomes *y*_0_ or *y*_1_. Then, given a data set *D* = {(*x*_1_, *y*_1_), …, (*x*_*N*_, *y*_*N*_)} of observations we can calculate the posterior probability over functions *f*:

$$p\left(f|D\right)=p\left(f\right)\frac{p(D|f)}{p(D)}$$

Thus, instead of inducing a probability distribution over parameters as in a linear regression, the dataset will induce a probability distribution over functions themselves.

We now need to specify a prior over functions *f* (note that the functions are random variables in this context) of the running variable *X*, and GPs derive from the use of Gaussian priors. Thus *p*(*f*) is a GP if for any subset of values of the running variable *x*_*i*_⋯*x*_*k*_, the joint distribution of the corresponding outcome functions *f*(*x*_*i*_), ⋯*f*(*x*_*k*_) is a multivariate Gaussian. GPs are parameterized by the mean of the GP *μ*(*x*) and its covariance function or Kernel *K*(*x*, *x*′). For pairs of *x*_*i*_, *x*_*j*_ we thus have

$$p\left(f\left(x_{i},x_{j}\right)\right)=N\left(m,\Sigma \right)$$

where

$$m=[\begin{array}{c} m(x_{i})\\ m(x_{j}) \end{array}]$$

and

$$\Sigma =[\begin{array}{cc} K(x_{i},x_{i}) & K(x_{i},x_{j})\\ K(x_{j},x_{i}) & K(x_{j},x_{j}) \end{array}]$$

For the canonical choice of *m*(*x*) = 0 and a *K* that increases with distance, this induces a prior over functions *f* that have varying smoothness aspects [31].

A very popular choice is the squared exponential kernel:

$$\begin{array}{c} K\left(x_{i},x_{j}\right)≔\sigma_{f}^{2}\text{exp}(-\frac{\|\left(x_{i}-x_{j}\right){\|}^{2}}{2\ell^{2}}) \end{array}$$

The parameters of the squared exponential kernel are the variance $\sigma_{f}^{2}$, which formalizes how much the function varies around each mean, and the spatial scale *ℓ*, which formalizes how quickly the function can change when *x*_*i*_ and *x*_*j*_ get further apart. The larger *ℓ* the lower the probability of non-smooth functions. Once mean and kernel functions are defined, we can fully define the Bayesian model:

$$y_{i}=f\left(x_{i}\right)+\varepsilon_{i}$$

where *f* ∼ *GP*(0, *K*), $\varepsilon_{i}∼N\left(0,\sigma_{i}^{2}\right)$

The likelihood of a GP is Gaussian, so the posterior is also a GP. We can, therefore use the properties of the multivariate Gaussian distribution to make predictions for the value of the outcome y_*_ at any covariate location x_*_:

$$p\left(y_{*}|x_{*},D\right)=\int p\left(y|x_{*},f,D\right)p\left(f|D\right)df$$

In the context of RD designs, GPs can be used to describe the potential outcomes as a function of the running variables [30]:

$$Y_{i}\left(0\right)=\mu_{0}\left(x_{i}\right)+\varepsilon_{i0},\;\varepsilon_{i0}∼N\left(0,\sigma_{0}^{2}\right)$$

$$Y_{i}\left(1\right)=\mu_{1}\left(x_{i}\right)+\varepsilon_{i1},\;\varepsilon_{i1}∼N\left(0,\sigma_{1}^{2}\right).$$

The conditional expectations *μ*_0_(*x*_*i*_), *μ*_1_(*x*_*i*_) are assumed to be independent. Thus, a separate model can be fit independently on each side of the threshold. Using the same kernel in both priors corresponds to assuming equal covariance matrices for treatment and control, and is similar in nature with fitting LLR with different slopes and intercepts but with the same bandwidth. The LATE is then the difference of the corresponding posteriors:

$$\begin{array}{c} \hat{\pi }\left(c\right)=\mu_{1}\left(c\right)\left|x,y-\mu_{0}\left(c\right)\right|x,y. \end{array}$$

To identify the optimal threshold for the utility function, we need to extrapolate the posterior functions to obtain an estimate of LATE at any covariate value *x*:

$$\begin{array}{c} \hat{\pi }\left(x\right)=\mu_{1}\left(x\right)\left|x,y-\mu_{0}\left(x\right)\right|x,y, \end{array}$$

and then numerically solve the optimization problem (i.e. find a root for $\hat{\pi }\left(x\right)$ near *c*).

GP extrapolation is also called Kriging and we know much of the properties of this estimator [32, 33]. GPs implement many intuitions that we have about functions in many real world situations, such as smoothness of the function.

## 5 Risk averse threshold optimization

In many cases, we are risk averse. Moving the threshold to the value that maximizes the expected utility implies moving to an expected zero local average treatment effect with some uncertainty. However, we may not want to risk a negative LATE. More specifically, we may be unwilling to accept a new threshold if its LATE has considerable probability of being negative. After all, that would mean that there are a range of values of the running variable where the treatment is actually harmful. Such a risk-averse objective may be particularly relevant in domains in which the use of RDD approaches is not yet established, e.g. in medicine [34].

In this case, we want to move to a threshold that is into the right direction (i.e. towards maximizing the utility function) but guarantees, with some confidence, that it will not produce harm, i.e. it will not result in a negative LATE. A natural way of implementing this, would be to use a threshold *c*_*α*_ which corresponds to the value for which the lower bound of the one-sided (100 − *α*)-confidence interval for $\hat{\pi }\left(x\right)$ is equal to 0. If $\sigma_{\hat{\pi }\left(x\right)}$ is the standard deviation of $\hat{\pi }\left(x\right)$ as a function of the threshold, this threshold satisfies

$$\begin{array}{c} \hat{\pi }\left(x\right)-Z_{\alpha }\sigma_{\pi (x)}=0, \end{array}$$ (5.1)

where *Z*_*α*_ is the critical value of the one tailed *Z*-test at level *α*. Based on the estimation procedure for $\hat{\pi }\left(x\right)$, we can obtain standard errors and confidence or credible intervals. Note that one may also want to use robust estimates, see [18]. The threshold for which Eq (5.1) holds can then be solved numerically.

**Fig 2:**
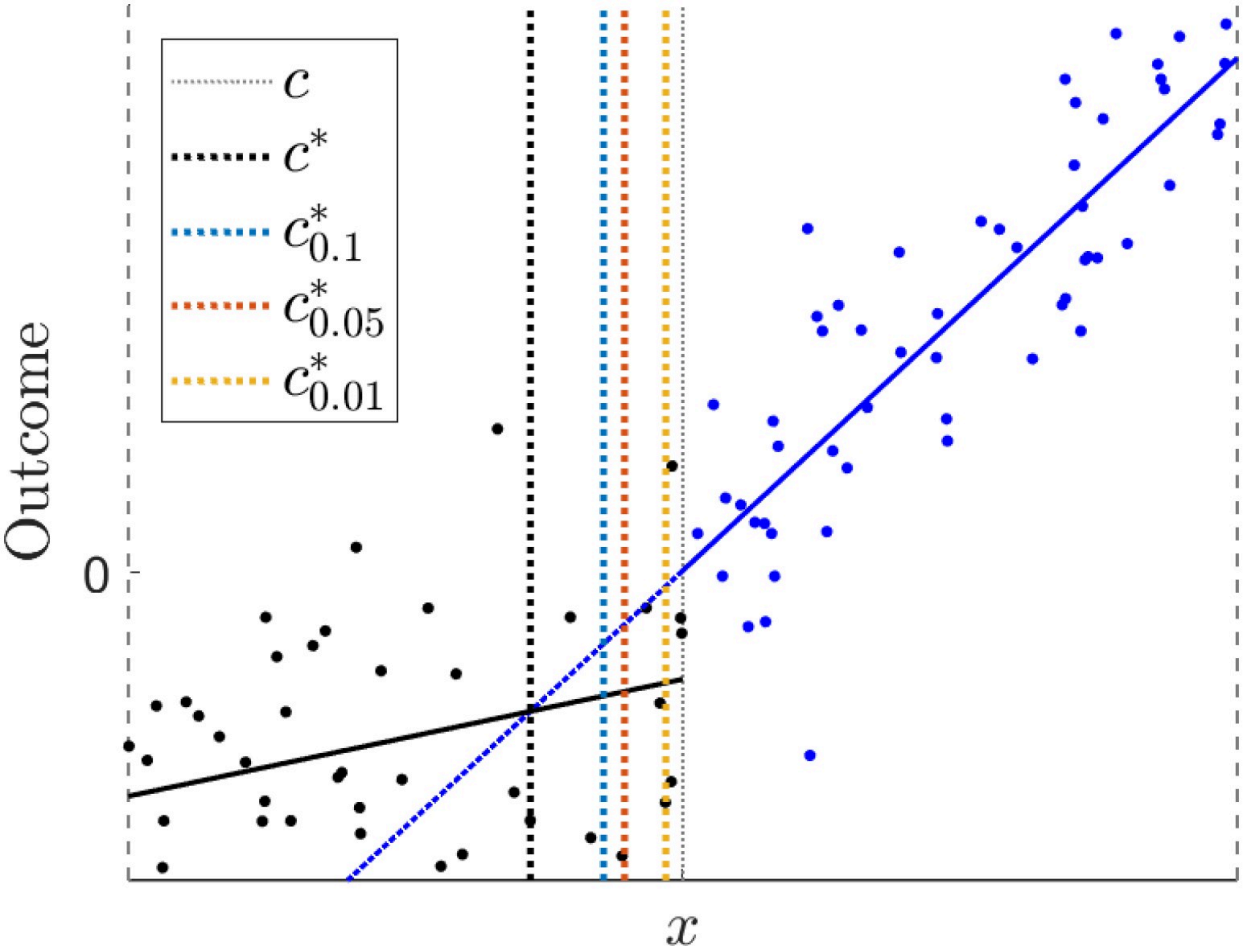
An example of conservative threshold estimation for a risk-averse policy, for 100 data points simulated from the model in Fig 1 (left). Using the (100 − *α*)%-confidence intervals for *π*(*x*), we can calculate conservative optimal thresholds $c_{\alpha }^{⋆}$, that improve the utility of the intervention, but guarantee with a probability of (100 − *α*)% that the LATE $\pi (c_{\alpha }^{⋆})$ will be non-negative.

Being risk averse results in smaller threshold changes. Fig 2 shows an example. Fig 2(a) shows 100 data points simulated from the model shown in Fig 1(left column) and the corresponding fitted LLR models left and right. Coloured vertical lines indicate the predicted optimal threshold for various confidence levels.

For smaller sample sizes or large variances, it is possible that the threshold cannot be moved at all. However, if we have a LATE estimate that is significantly larger than zero at level *α*, we can always lower the threshold to a value that results in a non-zero LATE with (100 − *α*)% probability and a larger utility value.

## 6 Threshold optimization with costs constraints

Most policies result in a cost per subject treated. Assume that treatment of each subject costs a fixed amount *z*. In the case of the linear regression (section 4.1), the optimal threshold is no longer for $\hat{\pi }\left(x\right)=0$, but for $\hat{\pi }\left(x\right)=z$, reflecting the condition that marginal cost equals marginal benefit. This yields

$$\begin{array}{c} \hat{c^{*}}=c-\frac{\left({\hat{\alpha }}_{1}-z\right)-{\hat{\alpha }}_{0}}{{\hat{\beta }}_{1}-{\hat{\beta }}_{0}}. \end{array}$$ (6.1)

Typically, policy implementations are subject to additional constraints related to the budget for the intervention, so that the treatment cost for all treated subjects cannot exceed a given budget *B*. In that case, for an *N*-sized population (including treated and controls), finding the optimal threshold corresponds to the following constrained optimization problem:

$$\begin{array}{l} \underset{t}{\text{maximize}}\;\;c^{*}=\underset{t}{\text{arg}\;\text{max}}\int_{t}^{c+h}\pi (x)p(x)dx,\\ \text{such}\;\text{that}\;\;N\int_{c*}^{x_{max}}p(x)dx\leq B, \end{array}$$ (6.2)

Without additional assumptions about the population density *p*(*x*), Problem (6.2) is a non-linear constrained optimization problem with non-linear constraints. The problem can be solved numerically, using any parametric or non-parametric density estimator for *p*(*x*).

Other types of constraints are also possible. For example, a prescribed drug may have adverse effects that are also related to the running variable. Depending on the problem-specific cost constraints, the constrained threshold optimization problem can be solved analytically or numerically.

## 7 Case study

We use our approaches on an example from the economics literature, to show how we can estimate the optimal threshold for an intervention. We use the sharp RD design from [23], who study the effect of default options in consumer choices. The authors use data from all New York City yellow cab rides in 2009; summary statistics for the data can be found in Table 1. During this period, customers of a specific credit card machine company (vendor) were offered different default tip suggestions based on the the cab fare. Specifically, the default tip suggestions for fares under $15 were $2, $3, and $4. For fares over $15, the tip suggestions were percentages of the fare amount: 20%, 25%, and 30%. At the $15 discontinuity, this change of policy corresponds to an increase of the suggested amounts by respectively $1, $.75 and $.5. Assuming that features that affect tips vary smoothly with the ride fare, the average difference of tips right and left of the margin can be interpreted as a result of policy change, i.e. higher default tip suggestions at the margin.

**Table 1:** Summary statistics for the data used in the case study. Standard deviations are in parenthesis. The sample is limited to rides without tolls, taxes, or surcharges (January 1,2009-January 31, 2009; 6 am-4 pm on Monday-Friday and 6 am-8 pm on Saturday and Sunday), as described in [23]. Data were downloaded from https://www.aeaweb.org/aej/app/data/0603/2013-0098_data.zip.

Summary statistics		
	T = 0 (Fare < $15)	T = 1 (Fare > $15)
Fare	8.727	18.419
	(6.506)	(6.888)
Tip amount as percentage of fare	21.1853	17.054
	(177.050)	(80.416)
Num. observations	453371	54269

[23] used local linear regression with a bandwidth of $10 around the discontinuity, and found that the local average treatment effect for cab fares of $15 was an increase of approximately $0.30 in tip amounts. This constitutes an increase in the average tip at the margin of more than 10%.

Optimizing the threshold in this case study can allow us to maximize the average tipping over all customers that receive the increased tipping suggestions. The positive effect at the discontinuity suggests that the policy threshold should be moved to the left, i.e. people with lower cab fares should also receive percentage tip suggestions. The original data set consists of 13,820,784 data points. To make the problem tractable, we used the data from all rides in January of 2009 with the specified vendor, resulting in 453,371 rides that cost $5—$15, and 54,269 rides that cost $15—$25. We used both local linear regression (LLR) and Gaussian Process regression (GPR) to model the conditional expectations *μ*_1_(*x*) and *μ*_0_(*x*) left and right of the threshold.

**Fig 3:**
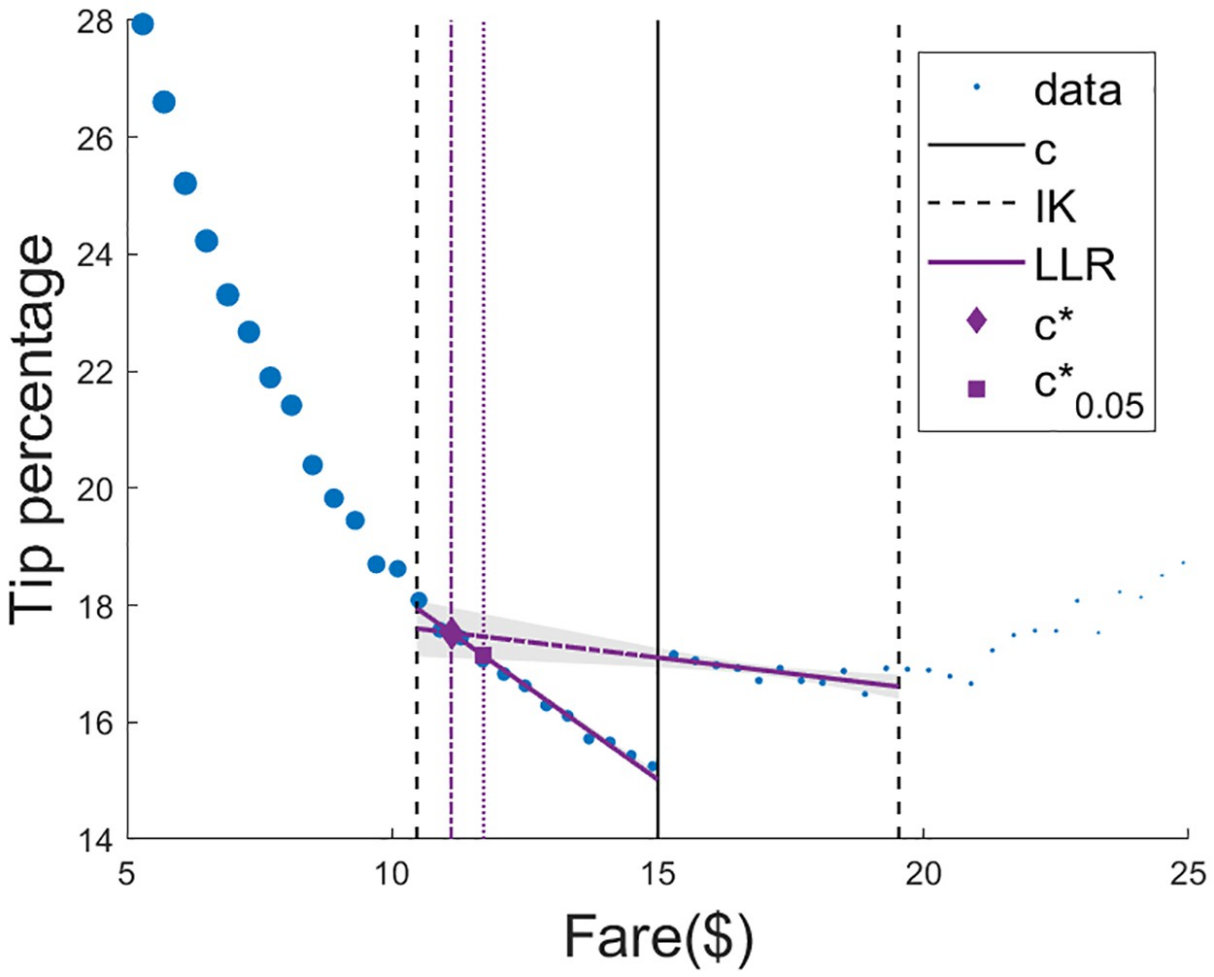
Using local linear regression to predict the optimal threshold for changing default tip suggestions in taxi cabs. Each dot is the average within a discrete fare amount ($0.40 intervals). Dot size is proportional to the number of observations in that interval. Policy for default tip suggestions changes for $15 rides. Imbens-Kalyanaraman (IK) bandwidth was estimated at $4.54 around the threshold. LLR models were fit left and right of the threshold with in the IK bandwidth. The optimal threshold is at $11.11, and the conservative threshold for significance level *α* = 0.05 is at $11.72.

We used LLR with the bandwidth selection method of [24] a uniform kernel. The optimal bandwidth selected by this method was 4.54. LLR with a uniform kernel were estimated on both sides of the threshold. The optimal threshold *c*^⋆^ was calculated using Eq (4.1) at $11.11 (see Fig 3).

The overall gain for drivers from switching to the optimal threshold corresponds to the expected gain in tip percentage:

$$\int_{c^{⋆}}^{c}({\hat{\mu }}_{1}\left(x\right)-{\hat{\mu }}_{0}\left(x\right))p\left(x\right)dx.$$

Using the discretization bin of $0.40 in the interval [*c*^⋆^, *c*], we calculate *p*(*x*_*i*_), the frequency of observations in each bin, where *x*_*i*_ are the discretized values of the running variable. The expected increase in tip percentage is then the finite sum $\sum_{i}({\hat{\mu }}_{1}(x)-{\hat{\mu }}_{0}(x))p(x_{i})=0.90$ percentage points. This corresponds to an expected gain of $\sum_{i}({\hat{\mu }}_{1}(x)-{\hat{\mu }}_{0}(x))p(x_{i})x_{i}=\$0.12$ perride. Thus, the method suggests that, by choosing the optimal threshold of $11.11 for switching default tip suggestions, taxi drivers would have earned, in total, an additional $11,011 over 89, 805 rides with rates within [$11.11, $15] in the month of January.

We want to note though, that there may be a negative side effect of suggesting large tip amounts: disgruntled customers, who may end up tipping less than predicted by our extrapolation. This would push us to be a bit more conservative. The conservative optimal threshold $c_{0.05}^{⋆}$ satisfying Eq (5.1) for *α* = 0.05 was estimated using MATLAB’s default root finding function [35] at $11.72. The expected increase in tip percentage is 1.17 percentage points, corresponding to an additional $0.16 per ride. The gain per ride is higher for the conservative optimal threshold because we only include ranges of the running variable where the gain is high enough to avoid the risk of a negative LATE. On the other hand, the overall additional expected gain is lower with the conservative optimal threshold, at $10, 409 over 64, 489 rides with rates within [$11.72, $15] in the month of January. (Fig 3). Table 2 shows bootstrap estimates and standard errors for *c*^⋆^ and $c_{0.05}^{⋆}$.

**Table 2:** Optimal thresholds using LLR and GPR. To estimate IK bandwidths and LLR coefficients, data were re-centered around the discontinuity *c* = 15. For both methods, bootstrap estimates were calculated over 100 bootstrap samples. Bootstrap standard errors are in parenthesis.

Optimal thresholds					
		IK bandwidth	LATE	*c* ^⋆^	$c_{0.05}^{⋆}$
LLR	All data	4.536	2.086	11.109	11.721
	Bootstrap	4.280	2.058	10.829	11.596
		(0.576)	(0.112)	(0.777)	(0.502)
GPR	All data		2.017	12.048	
	Bootstrap		1.492	14.172	
			(0.783)	(2.458)	

**Fig 4:**
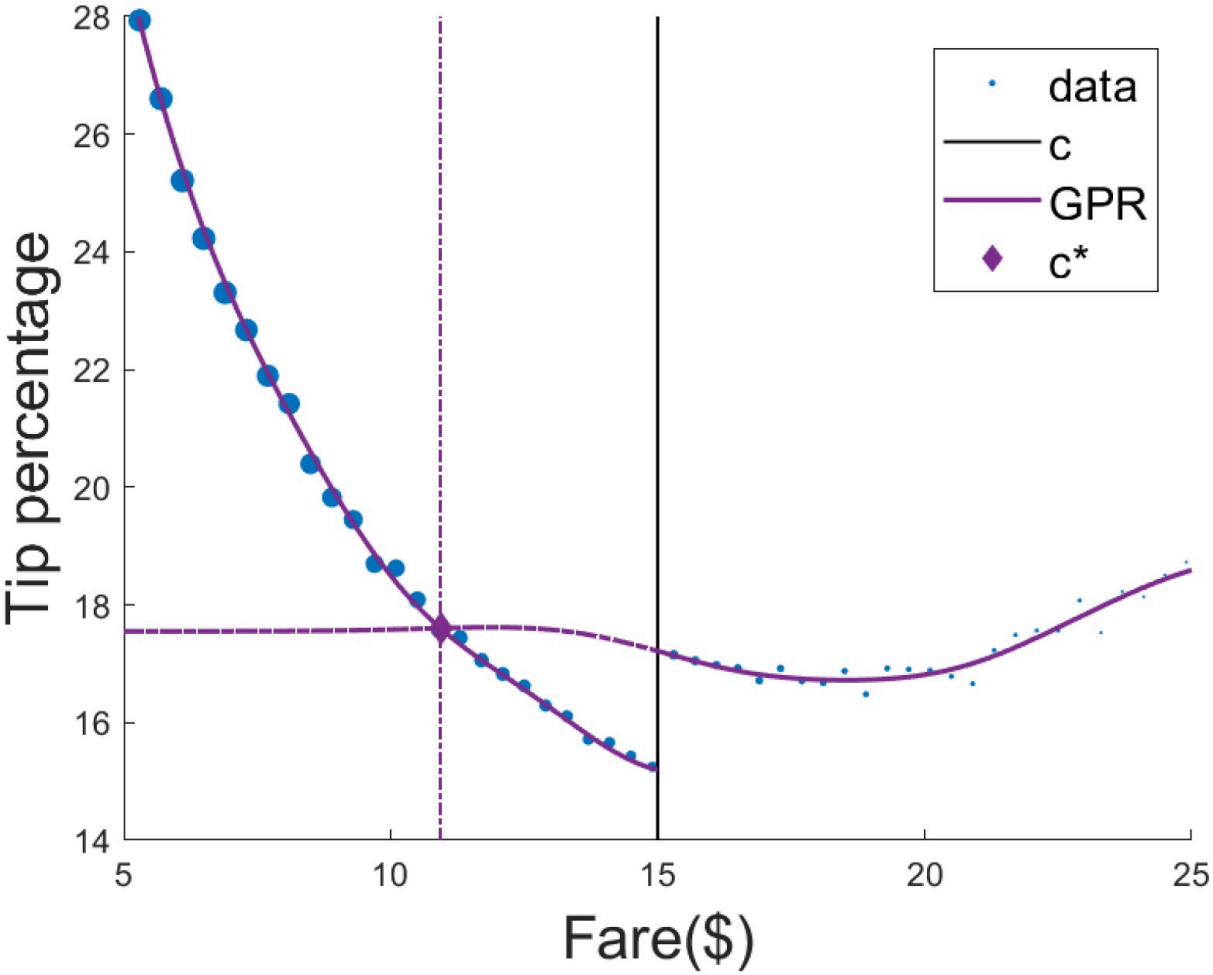
Using Gaussian process regression to predict the optimal threshold for changing default tip suggestions in taxi cabs. Each dot is the average within a discrete fare amount ($0.40 intervals). Dot size is proportional to the number of observations in that interval. Policy for default tip suggestions changes for $15 rides. Gaussian process regression was fit on the original bandwidth, using a double exponential kernel with parameters optimized using cross-validation. The optimal threshold is at $12.05. Due to large sample size, we were unable to obtain standard errors and estimate the conservative optimal threshold.

We also used GPR with the original bandwidth used by [23]. We used the double exponential kernel, with hyper-parameters optimized using cross-validation on each side of the threshold. The optimal threshold was estimated using MATLAB’s default root finding function [35]. Optimization predicts that, to maximize average tipping across the bandwidth, the default tip suggestions should switch to percentages for rides above c^⋆^ = $12.05 (Fig 4). Due to the large sample size, we were unable to perform exact inference and obtain standard errors for the fit, and therefore do not include a conservative threshold prediction for Gaussian process regression. Moreover, due to the large sample size, we used subsampled bootstrap of 10,000 observations per repetition to compute bootstrap estimates and standard errors for LATE and c^⋆^. In each sample, we again used the double exponential kernel, with hyper-parameters optimized using cross-validation on both sides of the threshold. Results are shown in Table 2.

Overall, both methods produce similar results. Moreover, the conservative threshold is really close to the unconstrained optimal threshold, due to the (unusually) large sample of the case study. Both methods suggest that, to maximize average tipping within the bandwidth, the default tip suggestions should switch to percentages for cab fares above $11.11 − $12.05 instead of $15.

## 8 Discussion and conclusion

Here we have shown how RD design can be exploited not only to estimate the local average treatment effect, but also to optimize the threshold itself. We proposed threshold optimization using local linear regression and Gaussian process regression, as well as conservative threshold optimization for situations where we are risk averse.

Most importantly, we show that RD designs with zero (or not statistically significant) estimates do not necessarily indicate ineffective policies. On the contrary, such interventions can correspond to policies implemented at the optimal threshold that maximizes welfare.

Optimizing the policy implementation threshold requires the assumption of policy invariance, i.e. that the LATE given the running variable does not depend on the threshold at which the policy is implemented. While there are cases where the assumption is violated, it is often satisfied in domains like medicine, where threshold optimization may be of great interest.

Given policy invariance, threshold optimization ultimately boils down to curve-fitting and extrapolation, since we are trying to guess the value of the LATE for unobserved regions of the running variable. Thus, any method is subject to modelling assumptions beyond the threshold.

Threshold optimization naturally implies that a meaningful welfare function exists and it is adequately measured. For example, if we want just want to maximize expected earnings without regard to any costs, our method can be applied straightforwardly as long as earnings data is available. However, in other cases, we have to provide as an outcome a summary measure that includes both costs and benefits. In many scenarios, e.g. educational policy [36], defining the utility function to be maximized is hard, since benefits and costs accrue to different entities. Consider the problem of student lifetime earnings versus cost of education to the university: should a university persist in educating students with low GPAs? Even though an RDD estimation shows that the earnings effects of allowing a few more students to stay enrolled can be positive [37], should the university spend a dollar for every extra dollar these students make? In such cases, constrained threshold optimization that abides to cost constraints could be explored. On the other hand, there are many domains where there is a consensus on what outcome we should maximize. For example, maximizing quality of life adjusted years is a standard goal in health care. In these cases, exploiting RD structure to optimize policy thresholds can have a straightforward and significant impact on policy design. While defining a meaningful welfare function can be difficult in some cases, specific welfare functions are well accepted by the community of experts in many domains.

Interestingly, the optimization approach could be reversed. If we are, for example, interested in the motivations of a politician (or decision-making body), we might want to ask for which social welfare function the actually chosen threshold is optimal. This might give evidence, for example, about how much a politician weights the potential future earnings of disadvantaged students relative to the immediate cost of tax increases to fund public university education.

Direct threshold optimization problems abound. In medicine, countless thresholds guide physician behavior. These could all be optimized for outcomes. In finance, thresholds that affect access to financial products by customers could be optimized. For internet companies, the probability of someone clicking ads can be optimized. And, in public policy, the various program thresholds could also be optimized. There are many domains where optimizing thresholds in the context of an RDD seems highly promising.
